# Conveying Safety Messages on Agricultural Machinery: The Comprehension of Safety Pictorials in a Group of Migrant Farmworkers in Italy

**DOI:** 10.3390/ijerph16214180

**Published:** 2019-10-29

**Authors:** Giorgia Bagagiolo, Lucia Vigoroso, Federica Caffaro, Margherita Micheletti Cremasco, Eugenio Cavallo

**Affiliations:** 1 Institute for Agricultural and Earthmoving Machines (IMAMOTER), National Research Council of Italy (CNR), 10135 Torino, Italy; g.bagagiolo@ima.to.cnr.it (G.B.); l.vigoroso@ima.to.cnr.it (L.V.); eugenio.cavallo@cnr.it (E.C.); 2 Department of Life Sciences and Systems Biology, University of Torino, 10123 Torino, Italy; margherita.micheletti@unito.it

**Keywords:** agricultural accidents, migrant workforce, occupational safety, risk communication, pictorials

## Abstract

The comprehension of safety signs affixed to agricultural machinery is fundamental to warning users about the residual risks which cannot be eliminated with machinery design and the adoption of protections. This is particularly relevant for the migrant workforce, which may encounter some language barriers with written safety communication. The present study aimed to investigate the comprehension of safety signs affixed to agricultural machinery in a group of migrants from both European and non-European countries employed in Italian agriculture. Thirty-seven migrant farmworkers (12 Indians, 17 Pakistanis, and eight Romanians) were individually interviewed to test the comprehension of four safety signs referring to the main causes of fatal and non-fatal injuries caused by interactions with farm machinery. Romanians obtained the highest comprehension performance (68.8% of correct answers), followed by Indians (35.4%), with Pakistanis being last (32.4%). The nationality and the previous experience as a farmworker significantly affected the comprehension of safety signs. The results pointed out the importance of adequately training migrants on the meaning of safety signs. Beside this, the study suggests a redesign of the signs, considering some signs’ features to enhance pictorials’ cross-cultural comprehension.

## 1. Introduction

### 1.1. Risk Communication in the Workplace

Communicating risks in the workplaces is important to preventing injuries [1], especially in those sectors where the interaction between humans and hazardous machinery takes place every day. Safety signs are widely used for this purpose [2]. The graphical elements depicted on a safety sign, usually named pictorials, have the main purpose of informing workers about health and safety issues, warning the users against existing hazards and suggest how to avoid them [3]. Regarding machinery, the last step of the safety hierarchy protocol in machinery design [3] specifies that pictorials shall be adopted to warn users against residual risks from the machinery which cannot be eliminated with design and the adoption of protections [3,4]. Compliance with such safety hierarchy protocol has been mandatorily introduced by the European Union (EU) for the machineries marketed in its countries, to indirectly improve occupational safety [2]. Considering pictorials’ key role in providing safety information, many standards and regulations [5,6] establish common guidelines for the design of safety pictorials, to make them universally comprehensible [7], including among illiterate and non-native language users [8]. A relevant challenge regarding safety pictorials comprehension is provided by migrant workers, who represent a high percentage of the workforce in the high-income countries [9]. Migrant workers are particularly prone to accidents and injuries compared to local workers [10]. Thus, it is fundamental that safety signs, as a risk prevention tool, are well comprehended among different ethnic groups.

Contrary to the expectations, clearly communicating a message by means of safety pictorials is not easy [11]. Indeed, previous studies in the pharmaceutical and medical [12], pesticide [13], traffic [14] and construction [15] sectors, show that some pictorials are clearer to understand without any explanations, while others are harder to comprehend [16,17] and different user factors appeared to play a role in pictorials’ comprehension. Contrasting results are reported, in particular, in regard to age, education, and previous experience. In some studies, the age and educational level of the target audience seemed to influence the comprehension of pictorials, with younger [18] and educated [19] participants reporting a better comprehension [20,21], whereas other studies found no significant main effects of these variables [19]. Additionally, in regard to the influence of previous experience, contrasting results are reported in the literature. In particular, a study by Liu et al. [14] reported no significant changes in safety sign comprehension for participants who were familiar with machinery, while on the other hand, a number of studies remarked on the influence of previous exposure to pictorials on comprehension performance [22,23]. Studies involving groups of participants from different cultures [24] showed that people from different countries gave different interpretations of the same symbol, threatening the supposed cross-cultural comprehensibility of pictorials [25].

Pictorial comprehension has been under-investigated in the agricultural sector. Agriculture is indeed one of the most dangerous work sectors, with higher risks of fatal and non-fatal accidents, due to the presence of potentially dangerous machinery, hazardous substances, and dangerous environmental conditions [26,27]. In particular, machinery has been recognized as the main cause of workers’ injuries, mainly because of crushing caused by tractor rollover, cutting from rotating parts of the machinery, accidents during machinery maintenance, and falls occurring when mounting or descending a vehicle [28,29,30]. In addition, agriculture employs a high percentage of migrants in the high-income countries: according to the International Labour Organization (ILO) [10], 16.7 million migrants work in the agricultural sector and they are highly exposed to safety risks compared to local workers [31].

### 1.2. Context and Aim of The Present Study

According to the latest statistics, almost 600,000 migrants in Italy are employed in agriculture; most of them are European Union citizens, especially being from Romania [32]; however, the rate of workers coming from non-EU countries is increasing. In addition, a high turnover of migrant farmworkers is reported, due to the high percentage of seasonal employment, especially in the north of the country [33], where foreign workers represent between 60% and 75% of the seasonal contracts. Analysing the Italian farming population by nationality, it has been observed that the highest number of non-EU farmworkers comes from India (18.5%). Agriculture is the sector where most, nearly 2/3, of the Indian migrant population in Italy is employed [32]. Pakistanis represent the second ethnicity among the migrant farm workforce; 24% of the Pakistani population in Italy is employed in the agricultural sector [33]. In Italy, Indian and Pakistani migrants are employed primarily in livestock farming, probably due to the fact that in their country of origin the primary economic activity is linked to the agricultural sector and the livestock heritage is remarkable. Migrant farmworkers in Italy represent a vulnerable population [34], and they are involved in hazardous situations and accidents more often than local farmworkers. Referring to the report of the Italian National Institute of Statistics (ISTAT), in the 2013, the migrants’ accident rate was 3.3%, against the 2.8% of local farmworkers [10].

Based on the relevance of pictorials for providing safety information and the high rate of migrant workforce in the Italian agriculture, the aim of the present study was to investigate the comprehension of safety pictorials in a group of migrant farmworkers from both EU and non-EU countries employed in the husbandry sector in Italy. Four safety signs developed by ISO and ANSI standards [6,35] representing the main sources of accidents in the interaction with agricultural machinery were used for the study.

## 2. Materials and Methods 

### 2.1. Participants

The study was carried out on a sample of thirty-seven both European and non-European migrant farmworkers, permanently employed in the husbandry sector, who were at least occasional users of agricultural machinery or used to work in close contact with machines. All participants were males. Migrants employed in the fruit-growing sector were discarded from the present study, since they are seasonally employed for the harvesting, sorting, and storage of fruit, and it turns out that they do not use agricultural machinery for those operations.

To be included in the selected sample, all participants had to have passed the mandatory test of knowledge and comprehension of Italian language requested by the Italian OSH (Occupational Safety and Health) regulation (Decreto Legislativo 81, 2008) [36], an application of the European Framework Directive on Health and Safety at Work [37]. Moreover, the participants had to have already attended the mandatory health and safety basic training required by the OSH rules to be employed in Italy, which includes the explanation of how to read pictorials and how to recognize them in a variety of contexts.

The research was carried out in Piedmont region (north-western Italy) in the Province of Cuneo. Given the high rate of EU and non-EU migrant farmworkers hired in breeding farms [38], the Piedmont region represents the Italian agricultural migrant workforce well. More specifically, the province of Cuneo is among the first Italian provinces in terms of migrants being hired in the agricultural sector, since more than 50% of the workforce are migrant farmworkers [39].

### 2.2. Instruments

Four pictorials from the ISO 11684:1995 standard [5] were used in the present study. They were a subset of those whose comprehensibility had been already investigated among local Italian [40] and American (US) farming populations [41]. They were hazard avoidance (i.e., presenting visual instructions on how a hazard should be avoided, as defined in ISO 11684:1995) pictorials affixed on agricultural machinery, and they referred to the main causes of fatal and non-fatal injuries among farming population; i.e., machinery maintenance, tractor rollover, entanglement, and cutting [30] (see Table 1). Since the study was intended to investigate the comprehension of graphical symbols, and following the method adopted by Caffaro, Mirisola and Cavallo [40], only the format with two panels, both illustrative and in vertical configuration was selected, with a safety-alert symbol above and the hazard avoidance pictorial below (see Table 1).

Each pictorial was printed on a paper sheet and shown to the participants in randomized order. The pictorials were presented in the same colour and size as recommended by ISO 11684:1995 [5] and ANSI Z535.3-2011 [6] standards: black drawings on a yellow background, 88 × 168 mm each. Following ANSI Z535.3 2011 guidelines, safety signs’ comprehension was assessed using open-ended questions in which participants were asked to describe the meaning of each symbol in their own words [42]. A sociodemographic form followed, to obtain data about personal and work characteristics; namely (i) age, (ii) years of education, (iii) length of stay in Italy (expressed in months), and (iv) previous experience as a farmworker in the country of origin. The study was conducted in accordance with the Declaration of Helsinki, and the protocol was approved by the Research Advisory Group (RAG) of the Institute for Agricultural and Earthmoving Machines (IMAMOTER) of the National Research Council of Italy (CNR) on November 15, 2016.

### 2.3. Data Collection Procedure

Participants were met at the farm where they were working and interviewed by the authors. Although the participants had passed the compulsory test of basic knowledge of Italian language, following the same procedure adopted by Smith-Jackson and Johnson [43], an interpreter supported migrants to understand the questions in case of trouble. The interpreter had been previously trained about safety risks in the agricultural sector and about the meaning of the safety pictorials to be shown to the participants during the interview. Based on the method adopted in other studies [43,44], each participant was individually interviewed and the responses were audio-recorded. After each participant gave his interpretation of all the four safety pictorials, the correct meaning was explained. The overall interview lasted between 20 and 40 min for each participant. Participation in this study was voluntary and no incentives were given. All the participants were informed on the nature of the study and their right to privacy was respected.

### 2.4. Data Analysis

Interviews were transcribed verbatim and then underwent a qualitative content analysis supported by NVivo software v.11 (QSR International, Melbourne, Australia). Participants’ responses were categorized into correct and incorrect answers, based on the intended meaning provided by ISO standard 11684:1995 for each pictorial (Table 1). Based on ANSI Z535-3:2011, correct answers included responses with intended meaning variations, in which symbols were defined not only in concrete terms but also conceptually; incorrect answers included answers that were wrong, no answer (7 cases out of 148 answers), or answers that were critical confusions (i.e., “When a safety symbol elicits the opposite, or prohibited action. For instance, when a safety symbol meaning "no fires allowed" is misunderstood to mean "fires allowed here"” [7] p.1). Following the scoring procedure provided by ANSI Z535-3:2011 [6], a correct answer was scored 1, whereas an incorrect answer was scored 0. Two independent judges coded the responses, reaching an agreement rate of 81%, and any disagreement was discussed until consensus was achieved. A total comprehension score for the four pictorials were then computed for each participant (ranging from 0 to 4, where 0 meant no correct answers for any of the pictorials and 4 indicated that each pictorial yielded a correct answer). 

Basic descriptive statistics as means, frequencies, and percentages were calculated, for both demographic characteristics and comprehension performance. An analysis of covariance (ANCOVA) with Bonferroni-adjusted post hoc tests was then performed to test the effects of user variables included in the study on the comprehension score. Cross-cultural differences were analysed by including nationality as the between-subject factor, whereas age and previous experience as a farmworker were included as covariates, following the literature review. Prior to analysis, diagnostic and normality tests were conducted. Scatter plots and histograms were generated and Shapiro–Wilk tests performed for the variables considered in the analysis (i.e., age, education, number of months living in Italy, and the total comprehension score). Number of months in Italy and the total comprehension score were not-normally distributed (W(37) = 0.862, *p* = 0.000 and W(37) = 0.910, *p* = 0.006, respectively) and they showed a positive skew of 0.850 (SE = 0.388) and 0.326 (SE = 0.388), respectively. Transformations were unsuccessful in achieving normality for these variables. However, adopting the same approach reported by Govindu and Babski-Reeves [45] and Caffaro et al. [46], and since the analyses used for the study are known to be robust with regard to normality assumptions [47], the data were used in their raw format. In order to control the independence of covariates from the nationality factor, a series of preliminary analyses of variance (ANOVA) were performed. No significant differences emerged either in regard to age (*p* = 0.879) or previous job (*p* = 0.315), while other demographic variables, such as years of education and length of stay in Italy, resulted in confounding variables, and for this reason they were not included in the subsequent analysis. The statistical analyses were computed using SPSS Statistics, v.23.0 (IBM Corp., Armonk, NY, USA).

## 3. Results

### 3.1. Sample Composition

Table 2 reports the composition of the sample involved in the present study, including Indian (*n* = 12), Pakistani (*n* = 17) and Romanian (*n* = 8) farmworkers. Such composition mirrors, by area of origin and gender, the actual composition of the workforce in the animal husbandry sector, that is comprised of mainly migrants from the Indian subcontinent and from Eastern Europe [38].

### 3.2. Comprehension Scores

The overall total scores of the frequency of correct answers are summarized in Table 3, while Table 4 illustrates the percentage of correct answers for each pictorial and each nationality. All the participants recognized the pictorial with the exclamation mark as a symbol of warning and the presence of some form of danger.

Concerning the comprehension of the lower panel of the safety signs, only two out of 37 participants (5.4%), one from India and one from Romania, gave a correct answer for all the four pictorials investigated, while five participants, one Indian and four Pakistanis (13.5%) did not give any correct answers (total comprehension score = 0). 

None of the four pictorials investigated obtained a comprehension level over 85%, which is the minimum level recommended by ISO and ANSI standards for a safety sign to be considered understandable [6,48]. The pictorial that yielded the highest comprehension score (Table 3) was the one representing the risk of tractor rollover (#4) (78.4% correct answers), followed by pictorials #3 (40.5% of answers correct), #1 (29.7% of answers correct), and #2 (16.2% of answers correct), referring to the risk of severing a foot, the need to consult technical manual, and the risk of entanglement, respectively.

Within each nationality, the same ranking was observed for each pictorial. Among Pakistani, nobody was able to give the correct meaning for pictorial #2. On the other hand, Indians obtained the best comprehension scores in pictorials #4 and #1, followed by pictorials #3 and #2. Romanians gave correct answers 100% of the time for pictorial #4, had 62.5% of their answers correct for pictorials #2 and #3, and 50% of their answers correct for pictorial #1. Table 4 shows the frequency of correct answers in relation to pictorials’ meanings, displayed by participants’ countries of origin.

### 3.3. The Effects of Migrant Farmworkers’ Variables on Comprehension Performance

The ANCOVA showed that there were significant differences in comprehension performance in regard to migrant farmworkers’ nationalities (F (2,36) = 9.30, *p* = 0.001). The highest overall mean of comprehension performance was obtained by Romanians (68.8%), followed by Indians (35.4%), and last were Pakistanis (32.4%). The difference between Romanians and others resulted in statistical significance according to Bonferroni post-hoc test (*p* < 0.05). 

In regard to the effect of other demographic variables, no statistically significant differences emerged for nationality when adjusted with the covariate age, while previous experience as a farmworker was shown to have a significant effect on comprehension performance (F (1,36) = 4.92, *p* = 0.03). Indeed, those participants who had an agricultural background showed higher comprehension scores.

## 4. Discussion

Since pictorial design is aimed to overcome literacy and language barriers, risk communication based on safety pictorials, particularly those affixed to agricultural machinery, represent a fundamental tool to reduce fatal and non-fatal injuries among migrant farmworkers. Despite a number of international standards [5,6,35] providing guidelines to make pictorials easily comprehended regardless of users’ characteristics, the outcome of the present study revealed that the migrant farmworkers comprehended the safety pictorials to some extent, but with high variability (correct answers ranging from 16.2% to 78.4%). Higher comprehension rates were obtained in previous research investigating the same four pictorials among local populations from Italy and the United States (reporting a comprehension rate ranging from 84.1% to 94.2% and 57.7% to 89.9%, respectively) [40,41], pointing out that these graphical symbols may fail in evoking universal comprehension amongst all users. In addition, the participants reported a high rate of incorrect answers despite the fact that they had previously attended a training course in which how to read and recognize safety pictorials was explained. This result may demonstrate the need for user-centred training programs [49,50], aimed at making visual communication instruments more noteworthy and recognizable by migrant farmworkers.

As concerns the comprehension score yielded by each investigated pictorial, the results showed that among the migrant farmworkers involved in the present study, some pictorials seemed to be easier to comprehend than others. In particular, the pictorials that reported the higher comprehension scores were pictorial #3 and pictorial #4, which represented, respectively, the risk of tractor rollover and the severing of foot due to rotating blades. Considering that vehicle overturning is one of the main causes of fatal injuries in agriculture [30], involving machines with on-board drivers [51] and operators [52], the high percentage of correct answers reported for this pictorial can be positively interpreted. Nevertheless, the need to achieve higher comprehension for pictorials yielding the lowest scores (#1 and #2, related to the need of reading the manual before performing any machinery maintenance and to the risk of entanglement in the power take-off, respectively) is particularly urgent, since these pictorials refer to some of the most severe causes of accidents as well [29].

Considering the cognitive features pointed out by Chan and Chan [53] which may affect users’ comprehensions of pictorials (namely, the connections of the object depicted with the real world, the amount of details represented, and the perceived meaningfulness of the pictorial) it could be noticed that in the present study, the higher percentages of correct answers were recorded for those pictorials with less details and which depicted a concrete action (i.e., rotating knives and tractor rollover); on the other hand, the lowest level of comprehension was yielded for those pictorials presenting no specific action and more details (i.e., consult technical manual and entanglement). Similar results are provided in the study conducted by Chan and Chan [53], in which the pictorial showing a worker who may get caught by machinery part (entangled), was perceived by participants “as the most complex, implying that the perceived simplicity of a sign was related to a number of the elements in the sign” [53], p.1502. In the same study, a pictorial in which a hand that held a wrench was depicted, reported lower levels of comprehension, due to a lack of information about the action for which the wrench had to be used. Regarding the representation of the targeted action, the results of the study conducted by Yamazaki and Taki [54] suggested that the representation of the object of the action (in our study, for instance, the tractor for the pictorial related to the need to consult technical manual) could be useful for increasing the comprehensibility of the pictorial. 

The issue related to pictorials’ features and their role in safety signs’ comprehension in different cultures should be further investigated, by identifying the most critical details for different cultures and by testing the efficacy of some redesigned solutions to enhance pictorials’ comprehension, as suggested by many authors [55,56]. For instance, considering the complex relationship between human figures and tools and machinery, it could be useful to: (i) reduce the number of additional details that could cause misunderstanding; (ii) depict the different parts of the whole pictorial improving the balance between solid shapes and outline shapes to make each part more comprehensible; and (iii) reduce the abstractness of the action, depicting the specific action.

In the present study, pictorial comprehension was affected by some characteristics of the target audience, such as farmworkers’ nationality and previous experience in the agricultural sector.

Considering nationality as a proxy of culture [57], the results of the present study are consistent with a number of previous works proving that cultural differences may impact a worker’s ability to understand images, and therefore, may affect comprehension of safety symbols and pictorials [15,23]. Indeed, Choi and Tay [58], noticed that it is fundamental to design safety signs considering the local context, since many standards were developed in different social and cultural environments which could affect users’ interpretation of the signs. Furthermore, Starren et al. [59] suggested to test pictorials among different cultures, because the information that pictorials communicates is limited, providing only evidence of a part of the risk, sometimes omitting information on how to prevent it, or the main cause of accident. 

In accordance with the previous study investigating migrant farmworkers’ interpretation of surround shapes around safety symbols [26], the present results highlighted that the migrants belonging to a ‘European culture’ (Romanians) reported the higher percentages of correct answers when interpreting the meaning of pictorials affixed to agricultural machinery. Very similar results were observed by Hare et al. [15] who investigated the effectiveness of safety signs among migrant workers employed in the construction sector in the United Kingdom. In their study, the nationality of the migrant workers resulted a significant predictor of their ability to comprehend and interpret the meaning of safety pictorials, and migrant workers from European countries correctly comprehended more pictorials than workers of African or Indian origin. More specifically, in regard to language and cultural differences, Kassam et al. [60] investigated the interpretation of pharmaceutical pictorials among linguistically diverse individuals belonging to a ‘non-European culture’ (Chinese, Punjabi Indians, and Somalis) and the results pointed out a generally greater difficulty in the comprehension of graphical representations based on ‘Western’ standards by non-European ethnic groups. According to Draffan et al. [61] the widespread use of “Westernized” symbols could highlight cultural differences and delay the recognition of a safety sign, while Blees and Mak [23] pointed out that symbols developed in one culture may not have the same meaning for people from another culture, and designers, maybe unconsciously, may adopt representational conventions which are more familiar for those participants who came from their same country. At the same time, the inadequacies of some kinds of pictorials in overcoming language barriers emerged. 

Besides the role of national culture in affecting the interpretation of safety pictorials, following literature suggestions, other co-variating characteristics of the investigated sample of migrant farmworkers were taken into account in the present study. In regard to prior experience as a farmworker when the participants were in their country of origin, the statistical analysis showed that this variable played a role in the correct interpretation of safety pictorials. This result is in line with the evidence from Huer [62], who suggested that culture/ethnicity and life experience have an impact on the perception of graphic signs. Previous experience as a farmworker can be related also to the ‘familiarity’ with the pictorials: indeed, it often happens that the more the sign had been seen previously during farm operation with machinery, the better the comprehension is; likewise, the greater the number of years of experience with agricultural machines, the better the comprehension, in line with the results obtained by Ng and Chan [19] about the positive effect of familiarity on traffic signs’ comprehension. No significant effects of age emerged, mirroring the results of previous studies [19].

### Limitations of the Study

Some limitations of the present study should be highlighted. First, a small, non-random sample of participants of different nationalities took part in the study, making the results non-generalizable. Migrant farmworkers represent a hidden and hard-to-reach population, which makes random sampling procedures rarely possible [63]. It is also not uncommon for studies addressing migrant workers to involve small groups of participants [43,64,65,66] due to the fact the migrant workforce is disseminated across the region and have varying operating schedules; thus, it is difficult to make a large and wide-ranging group of migrant workers converge. However, future research with larger sample sizes could help to reinforce the findings. Another limitation is related to the uneven distribution of participants among the different nationalities. Even though the overall composition of the sample reflected the general migrant agricultural workforce, a future development of the research with more balanced nationality categories would be welcome. Finally, we could not investigate the effects of education and length of stay in Italy, since in our study, those variables were not independent from the nationalities. As reported in previous literature, education may affect pictorials’ comprehension [19] and length of stay in a Western country may influence migrants’ degree of ‘Westernization’ and comprehension of safety information [57]. Future studies in which participants from different countries are matched based on their education level or length of stay in the host country will allow to assess the role played by these variables in pictorials comprehension. 

A possible development of the research could concern the issue of contextual information given with the pictorials. Considering the importance of contextual cues in pictorial comprehension [67], in the development of the literature it would be interesting to evaluate this variable more in depth, presenting to different groups of participants pictorials in their actual context of use; for instance, pictures of agricultural machinery on which these pictorials are affixed or scenarios of possible tasks for which each pictorial might be relevant, and pictorials without any contextual information.

## 5. Conclusions

Safety pictorials affixed to agricultural machinery have a great potential as a mean of communication, since they are supposed to convey messages about the residual risks in the interactions with machinery regardless of workers’ characteristics. Nevertheless, the results of the present investigation pointed out that pictorials were in general poorly comprehended by the migrant farmworkers, since none of the investigated pictorials obtained a comprehension level over 85%, the minimum level recommended by ISO and ANSI standards for a safety sign to be considered understandable. Besides that, some factors related to individuals’ characteristics, such as cultural background (nationality) and previous experience as farmworkers affected the interpretation of safety pictorials, thus questioning their effective cross-cultural and universal comprehensibility. In high-risk-related sectors such as agriculture, the lack of an effective risk communication by pictorials points out safety issues that are even more relevant, since the agricultural sector in developed countries employs a very high rate of migrant farmworkers, notably more exposed to fatal and non-fatal injuries compared to local workers. To face this issue, the results of the present study raised some considerations about the need to arrange a series of interventions tailored to migrant farmworkers and aimed at enhancing pictorials’ cross-cultural comprehension, including (i) testing pictorials among different cultures; (ii) adopting some technical re-design solutions that consider factors such as familiarity, simplicity, concreteness, and meaningfulness; and (iii) adequately training migrants with specific, user-centred programs finalized to achieve an effective risk communication among migrant workers. 

## Figures and Tables

**Table 1:** Safety pictorials and their meanings (from ISO 11684:1995).

P#	Pictorial	Description
#1	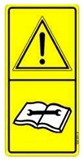	Attention—Consult Technical Manual for proper service procedures
#2	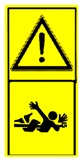	Attention—Whole body entanglement—Do not work on an installed power take-off with the engine running
#3	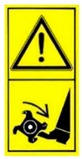	Attention—Severing of foot—Do not put feet under rotating knives
#4	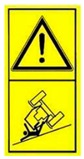	Attention—Tractor rollover—Do not drive on slopes where machine could slip or tip

**Table 2:** Demographic characteristics of the migrant farmworkers participating in the study.

Origin		Indians		Pakistani		Romanians	
		n	%	n	%	n	%
Previous experience as a farmworker	Yes	9	75	8	47	4	50
	No	3	25	9	53	4	50
		Mean (SD)		Mean (SD)		Mean (SD)	
Age (years)		34.4 (7.9)		32.6 (7.8)		32.8 (8.3)	
Education (years)		10.8 (1.3)		7.6 (2.2)		11.3 (2.7)	
Length of stay (months)		95.0 (44.2)		12.9 (4.2)		100.5 (29.4)	

**Table 3:** Comprehension scores for the four safety pictorials studied.

P#	#1	#2	#3	#4
**Pictorial**	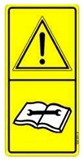	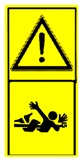	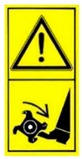	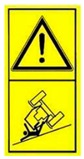
**Comprehension score ^a^**	**29.7%**	**16.2%**	**40.5%**	**78.4%**

^a^ Comprehension scores include the sum of correct answers given by the participant for each pictorial.

**Table 4:** Percentage of correct answers by nationality for the four safety pictorials studied.

Pictorial	Indians	Pakistani	Romanians
#	%	%	%
#1	41.7	11.8	50.0
#2	8.3	0.0	62.5
#3	16.7	47.1	62.5
#4	75.0	70.6	100.0
Mean (SD)	35.4 (0.3)	32.4 (0.3)	68.8 (0.2)

## Supplementary Materials

Supplementary File 1
