# Supplementary material for: Conveying Safety Messages on Agricultural Machinery: The Comprehension of Safety Pictorials in a Group of Migrant Farmworkers in Italy
Source: Int J Environ Res Public Health. 2019 Oct 29;16(21):4180. doi: 10.3390/ijerph16214180 (PMC6862054; doi:10.3390/ijerph16214180)
Supplement: Supplementary File 1 [file ijerph-16-04180-s001.pdf]

Consiglio Nazionale delle Ricerche
IMAMOTER
Istituto per le Macchine Agricole e Movimento Terra

Extracts of the Research Advisory Group meeting held on 15.11.2016

The IMAMOTER Research Advisory Group was established on 24th August 2015 (Regulation N. 2283) with the main purpose to define the scientific strategic directives of the Institute and promote the sharing of experiences and competences among the different research groups.

On November 15th, at 11 a.m., the Research Advisory Group of the Institute had a meeting at the headquarter of the IMAMOTER Institute in Ferrara. The meeting was chaired by the Director of the Institute, Ms Eleonora Carletti, and was attended by its members or in person or in videoconference.

-.-.-.-.-.-.-. OMISSIS -.-.-.-.-.-.-.-

The RAG examined the research proposal presented by Mr. Eugenio Cavallo titled: "Safety communication and migrant farmworkers: the role of visuals" aimed at investigating the comprehension of visual safety information among migrant farmworkers employed in the Piedmont region and identifying possible improvements. A correct comprehension of safety signs is indeed very important to prevent rollover accidents.

The applicant presented the whole proposal, the protocol of this study as well as the document for the informed consent to be delivered to all the participants of this study.

The RAG examined all the presented documents, carefully evaluated the feasibility of this proposal, every methodological aspect and the experimental design.

On the basis of the outcome of these evaluations, this research proposal was finally approved.

Ferrara, November 15th 2016

The IMAMOTER Director

Eleonora Carletti

Sede: Via Canal Bianco, 28 - 44124 FERRARA
Tel. 0532 735611 - Fax 0532 735666
U.O.S: Strada delle Cacce, 73 - 10135 TORINO
Tel. 011 3977 711/712 - Fax 011 3489218

info@imamoter.cnr.it
http://www.imamoter.cnr.it
C.F. 80054330586 - **P. IVA** 02118311006
